# A Skin Stress Shielding Platform Based on Body Temperature‐Induced Shrinking of Hydrogel for Promoting Scar‐Less Wound Healing

**DOI:** 10.1002/advs.202306018

**Published:** 2024-09-16

**Authors:** Qin Chen, Siyu Li, Ka Li, Weifeng Zhao, Changsheng Zhao

**Affiliations:** ^1^ College of Polymer Science and Engineering State Key Laboratory of Polymer Materials Engineering Sichuan University Chengdu 610065 China; ^2^ West China Hospital Sichuan University/West China School of Nursing Sichuan University Chengdu 610041 China; ^3^ Med‐X Center for Materials Sichuan University Chengdu 610065 China

**Keywords:** hydrogel wound dressing, mechanical transduction, skin scar, wound healing

## Abstract

Stress concentration surrounding wounds drives fibroblasts into a state of high mechanical tension, leading to the delay of wound healing, exacerbating pathological fibrosis, and even causing tissue dysfunction. Here, an innovative skin stress‐shielding hydrogel wound dressing is reported that makes the wound sites shrink as a response to body temperature and then remolds the stress micro‐environment of wound sites to reduce the formation of skin scars. Composed of a modified natural temperature‐sensitive polymer cross‐linked with polyacrylic acid networks, this hydrogel wound dressing has demonstrated a substantial decrease in scar area for full‐thickness wounds in rat models. The physical forces exerted by the wound dressing are instrumental in attenuating the activation and transduction of fibroblasts within the wound sites, thereby mitigating the excessive deposition of the extracellular matrix (ECM). Notably, the wound dressing significantly down‐regulates the expression of transforming growth factor‐β1(TGF‐β1) and collagen I, while concurrently exerting a dramatic inhibitory effect on the integrin‐focal adhesion kinase (FAK)/phosphorylated‐FAK (p‐FAK) signaling pathway. Collectively, the fabrication of functional hydrogels with a stress‐shielding profile is a new route for achieving scar‐less wound healing, thus offering immense potential for improving clinical outcomes and restoring tissue integrity.

## Graphical abstract

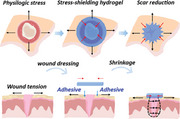

## 1 Introduction

Skin wound healing is a highly intricate and dynamic process that needs to activate and coordinate a large number of intracellular and intercellular signals to restore tissue integrity, preserve wound stability, and culminate in the formation of scar tissue.^[^ ^1^ ^]^ Unfortunately, various factors such as surgery procedures, trauma, burns, or other skin injuries inevitably result in the development of skin scars. These scars are characterized by excessive deposition, disrupted arrangement of extracellular matrix, and chronic inflammation, which significantly impact their appearance and function.^[^ ^2^ ^]^ Abnormal skin scars usually induce pruritus, redness, pain, and loss of limb mobility and skin function.^[^ ^3^ ^]^ Serious scars may break and fester over time, deteriorate, or even endanger life.^[^ ^4^ ^]^ Regrettably, there are no reliable measures for the prevention and treatment of skin scars. Consequently, skin scars continue to pose formidable challenges in the field of skin wound care.

Scars can be comprehended as a manifestation of tissue fibrotic disease resulting from incomplete skin damage repair from the perspective of molecular biology.^[^ ^5^ ^]^ Clinical studies have revealed that the areas characterized by substantial skin tension and stiffness, such as the joints, chest, upper back, and shoulders, are particularly susceptible to lesion formation.^[^ ^6^ ^]^ Hence, mechanical tension within the wound milieu is considered to be a pivotal factor affecting the formation of pathological fibrosis in skin histopathology. When the mechanical equilibrium between the periwound region and the tension‐bearing components of the wound is disrupted, mechanosensitive receptors are persistently stimulated, impeding proper tissue regeneration. Integrins, ion channels, G‐coupling proteins, and growth factors constantly activate cellular mechanoreceptors.^[^ ^7^ ^]^ Subsequently, the mechanical force signals are transformed into chemical information by molecules that are transmitted into the nucleus to affect the expression of mechanical force‐related genes for adaptation to the stimulation of external forces. Mechanical tension in the wound environment has been shown to drive fibroblasts toward an activated phenotype to differentiate into myofibroblasts, which accelerate the deposition of fibrotic collagen and continuously stimulate the biological processes of skin tissue repair, ultimately resulting in heightened scar formation.^[^ ^8^ ^]^ Worse still, a large amount of fibrotic tissue deposition will cause the deformation of the superficial structure and the structure of the dermis of the skin tissue. It is anticipated that scar‐free tissue repair will be possible if the steps are taken to balance the mechanical force between the periwound and tension‐bearing components in the wound environment, as well as the development of innovative mechano‐modulatory wound dressings.

Studies have reported that scar‐less wound healing happens during early in fetal gestation, which takes place in a low resting tension environment. Therefore, mechanical force in wounds has been proven to be a critical factor affecting skin scar healing.^[^ ^9^ ^]^ Previous attempts to mitigate tension in the periwound region involved the direct injection of botulinum toxin type A into the wound, inducing flaccid paralysis of the surrounding muscles and thereby reducing periwound tension and inhibiting scarring.^[^ ^10^ ^]^ However, this invasive approach and the associated pain caused by drug injection do not provide a good treatment experience for scar patients. In contrast, mechanical modulation presents a less invasive and painful alternative for scar patients. To facilitate wound contraction and alleviate tension, some researchers applied a polymer silicon sheet that was 40% pre‐stretched and then attached to the porcine wounds.^[^ ^11^ ^]^ However, uncontrolled contraction of the polymer silicone patch may cause the wound to be stressed in the direction of the patch contraction. Therefore, in the realm of wound care, maintaining a mechanical balance between biological skin tension and the load‐bearing aspects of the wound through the implementation of suitable dressings, is crucial for achieving favorable wound healing outcomes. Mechanical manipulation of injured or scarred skin has demonstrated promising potential for promoting skin healing and remodeling, as evidenced by both animal models and human clinical investigations.^[^ ^12^ ^]^ Nevertheless, current wound dressings used in standard care for skin wounds lack the capability to alter the mechanical microenvironment of the wound or manipulate tissue mechanics. Insufficient research has been conducted to explore the feasibility of employing mechanical modulation in skin wounds to reduce scarring.

From a clinical application standpoint, an optimal stress‐shielding wound dressing must satisfy two fundamental conditions simultaneously. The first requirement is that the dressing must have shrinkage mechanical properties that match wound healing, and the second is that the dressing should adhere to the skin wound under shrinkage conditions. Presently available wound dressings still lack the capability to form robust adhesion and active shrinkage on moist skin wounds.^[^ ^13^ ^]^ N‐isopropylacrylamide (NIPAm) is a material widely utilized for the preparation of autonomous contraction materials that respond to body temperature, owing to its low critical solution temperature that closely approximates normal body temperature.^[^ ^14^ ^]^ Li et al. prepared a series of multifunctional shrinkable hydrogel dressings with PNIPAm, quaternized chitosan, and reduced graphene oxide wrapped in polydopamine to repair skin defect tissues.^[^ ^15^ ^]^ Similarly, Hu et al. synthesized PNIPAm as the primary network within the hydrogel, enabling active contraction to accelerating the wound healing process.^[^ ^16^ ^]^ However, our previous research revealed a significant decline in adhesive properties of PNIPAm‐based hydrogels following temperature increase.^[^ ^17^ ^]^ This decline can be attributed to polymer chain associations that lead to the release of water molecules, resulting in the formation of a new hydration layer between the skin and the hydrogel dressing. Consequently, the reduced adhesive performance of the dressing hinders its ability to secure the wound edges and gather at the wound center to facilitate wound closure. Thus, designing a dressing that achieves rapid and robust adhesion to the wound skin while also possessing the mechanical contraction capability remains challenging.

**Figure 1:**
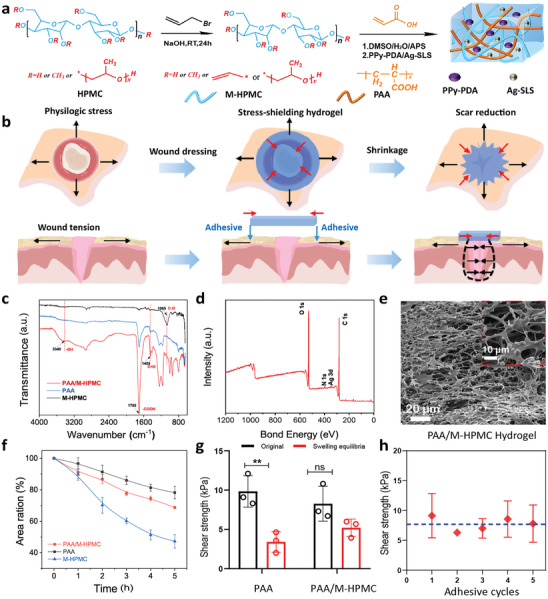
Schematic diagram of the synthesis and application of skin stress‐shielding platform hydrogel wound dressing. a) Synthesis of M‐HPMC and PAA/M‐HPMC hydrogel. b) Schematic illustration demonstrates that the active wound shrinkage enabled by stress‐shielding hydrogel to remold wound mechanical environment for reducing skin scar. c) FTIR spectra analysis of the PAA/M‐HPMC, M‐HPMC and PAA hydrogels. d) XPS spectrum of PAA/M‐HPMC hydrogel. e) Scanning electron microscope images of PAA/M‐HPMC hydrogel. f) The changes in the area of the PAA/M‐HPMC, M‐HPMC, and PAA hydrogels after incubating at 37 °C for 5 h. g) Comparison of shear strength between PAA and PAA/M‐HPMC placed on porcine skin before and after swelling for the lap‐shear test (The shear strength test of the M‐HPMC hydrogel was given up due to their lack of strength and toughness). h) Cyclic adhesion test of PAA/M‐HPMC hydrogel.

Motivated by the aforementioned insights, we established a skin stress‐shielding platform based on the modified hydroxypropyl methylcellulose (M‐HPMC) and polyacrylic acid, which possess temperature‐responsive contractility, skin tissue adhesiveness, antibacterial capability, and excellent biocompatibility. The schematic of the preparation process for this platform is shown in **Figure** **1**a. The M‐HPMC possesses a lower critical solution temperature (LCST) that triggers the changes in its hydration state in response to temperature stimuli, resulting in volume shrinkage. Moreover, the carboxylate group of the hydrogel facilitates the rapid absorption and removal of interfacial water, enhancing the adhesion between skin tissue and adhesive materials.^[^ ^18^ ^]^ In this design, a strongly adhesive hydrogel effectively grasps the edges of wound and generates contractile forces upon application to the skin. Given the inherent tension in the skin, the wound edges experience stress concentration, which can continuously stimulate the biological repair process, leading to increased scar tissue formation or hindering wound closure. Upon utilizing the skin stress‐shielding platform on wounded tissue, the hydrogel dressing performs adhesive property and is triggered by temperature change to generate contraction, thereby remolding the mechanical environment surrounding the wound. This skin stress‐shielding platform aims to achieve the goal of reducing or eliminating skin scars by reducing wound tension and influencing the interaction of collagen fibers and fibroblasts in newborn tissue. The schematic diagram of the stress‐shielding dressing to reduce skin scars is shown in Figure 1b.

In addition to its primary properties, the stress‐shielding platform possesses noteworthy secondary characteristics, including electrical conductivity (see Figure S1, Supporting Information) and antibacterial qualities. Our prior study demonstrates that conductive hydrogel doped with polypyrrole‐polydopamine (PPy‐PDA) nanoparticles has the ability to promote tissue healing.^[^ ^19^ ^]^ Furthermore, the incorporation of silver‐sodium lignin sulfonate (Ag‐SLS) nanoparticles into hydrogel dressings not only imparts resistance to skin infections but also triggers free radical polymerization reactions.^[^ ^20^ ^]^ Building upon these findings, we have developed a skin stress‐shielding dressing that integrates these concepts. To ensure the suitability of the hydrogel for use as a wound dressing material, we conducted a comprehensive characterization of its physical and chemical structure, as well as its functional performance. Subsequently, the hydrogel wound dressing was adhered to the full‐thickness defect wounds in rats to evaluate the efficacy of wound healing and scar formation. Various parameters such as scar area, color, ultimate tensile strength, and ECM structure of the scars were investigated. Additionally, immunohistochemistry staining analysis was explored to investigate the expression of FAK/p‐FAK mediated mechanical signaling and transforming growth factor TGF‐β1, α‐smooth muscle actin (α‐SMA) in animal tissue specimens.

## 2 Results and Discussion

### 2.1 Characterization of a Skin Stress‐Shielding Hydrogel Wound Dressing

Successful preparation of the M‐HPMC was confirmed by ^1^H NMR in Figure S2 (Supporting Information). Upon comparing the NMR spectra of M‐HPMC and HPMC, we found that the carbon–carbon double bond peak appeared between 5 and 6 ppm in the M‐HPMC sample. The FTIR spectra of the polyacrylic acid (yPAA)/M‐HPMC, M‐HPMC and PAA hydrogels are shown in Figure 1c. Notably, the characteristic peaks at 1065 and the 1702 cm^−1^ were identified as belong to M‐HPMC and PAA hydrogel, respectively. In the spectrum of the PAA/M‐HPMC hydrogel, we observed the presence of absorption peaks characteristic of both M‐HPMC and PAA. This outcome serves as evidence that the synthesis of the PAA/M‐HPMC hydrogel was indeed successful. Furthermore, XPS spectra indicated the composition of the PAA/M‐HPMC hydrogel, revealing the presence of C 1s, O 1s, N 1s, and Ag 3d (see Figure 1d). The morphologies of lyophilized PAA/M‐HPMC hydrogels were observed via SEM. As shown in Figure 1e, the PAA/M‐HPMC hydrogel exhibited an interconnected porous structure with complete pore walls. Notably, the pore size of the PAA/M‐HPMC hydrogel significantly differed from those of M‐HPMC and PAA, as observed in Figure S3a,b (Supporting Information), respectively.

We believed that a dressing could alleviate tension by bringing the wound edges closer together. To investigate this, we delved deeper into the behavior of skin tissue adhesion and contraction induced by temperature changes. Specifically, we studied the temperature‐responsive shrinking behavior of the skin stress‐shielding platform, PAA/M‐HPMC, and captured hydrogel images at 37 °C (Figure S4, Supporting Information). The area changes and shrinkage ratios of hydrogels were quantified, as shown in Figure 1f. After incubating all the hydrogels at 37 °C for 5 h, significant shrinkage occurred. However, the shrinking process of the PAA/M‐HPMC hydrogel occurred at a slower rate compared to the M‐HPMC hydrogel, resulting in a shrinkage rate of 31.2% after 5 h at 37 °C. Remarkably, only the PAA/M‐HPMC retained its tissue adhesion properties (see Movie S1, Supporting Information). To evaluate the adhesion performance of the PAA/M‐HPMC hydrogel patch on wounded tissue, we conducted a lap‐shear test using porcine skin as a model tissue (Figure S5a, Supporting Information) to measure the shear strength of the hydrogel. The shear strength refers to the force required to slide one surface parallel to the other in a direction parallel to the plane of the bond. The shear strength of the PAA/M‐HPMC hydrogel is 8.3 kPa, and its shear strength does not significantly decrease after absorbing wound exudate. This performance of the PAA/M‐HPMC hydrogel is clearly superior to that of the PAA hydrogel (Figure 1g; Figure S6, Supporting Information). To optimize the repeatable and durable adhesion performance of the PAA/M‐HPMC hydrogel, we demonstrated this concept by conducting cyclic lap‐shear testing over 5 cycles. A cycle consisted of attaching the hydrogel to the porcine skin surface, peeling it off by a tensile load (the PAA/M‐HPMC hydrogel remains intact and adheres to one piece of porcine skin, as shown in Figure S5b, Supporting Information), and then re‐adhering the same hydrogel before the subsequent cycle. The shear strength slightly decreases but does not affect usability, maintaining ≈7.5 kPa during all 5 cycles (Figure 1h). The adhesion capabilities of the skin stress‐shielding platform were found to be comparable to readily available tissue adhesives and wound dressing in the market, including cyanoacrylate adhesives (Dermabond), polyethylene glycol‐based adhesives (Coseal), fibrin‐based adhesives (TachoSil), and wound dressings (Tegaderm).^[^ ^13^ ^]^ Furthermore, the PAA/M‐HPMC hydrogel exhibited robust adhesion on various substrates, such as rubber, glass, plastic, steel, and foam (see Figure S7a, Supporting Information). Once adhered to porcine tissues, the stress‐shielding wound hydrogel transformed into a thin layer with tissue‐like softness and stretchability (see Figure S7b, Supporting Information). Notably, the adhered stress‐shielding platform could be detached from the tissue (see Figure S7c and Movie S2, Supporting Information). This gentle wound dressing platform could prove beneficial in clinical settings where frequent changes of wound dressings are necessary for wound healing management.

### 2.2 Temperature‐Responsive Behavior of Hydrogel

**Figure 2:**
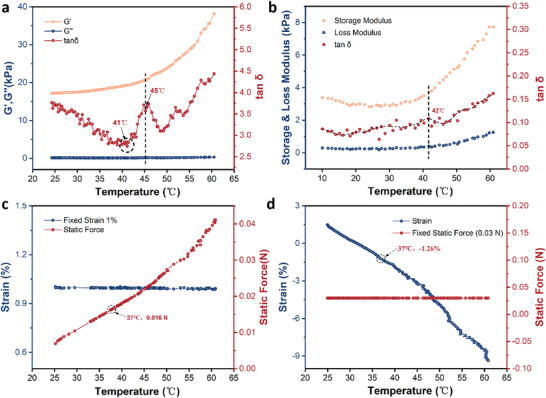
Temperature dependence of the dynamic modulus of PAA/M‐HPMC hydrogel a) rotational rheometer testing and b) dynamic mechanical analysis. c) The Static Force–Temperature variation curve under the condition of an initial strain of 1%. d) The Strain–Temperature variation curve under the condition of an initial static force of 0.03 N.

Proving that the PAA/M‐HPMC hydrogel has the ability to respond to temperature changes and quantifying the mechanical forces generated by the hydrogel during the contraction process is crucial for our research. To investigate the temperature‐responsive behavior of the PAA/M‐HPMC hydrogel, we conducted tests using a rotational rheometer and dynamic mechanical analysis. We performed a temperature sweep on the PAA/M‐HPMC hydrogel sample. As shown in **Figure** **2**a, it is evident that the storage modulus (*G*′) increases with rising temperature, while the loss modulus (*G*″) remains relatively unchanged. However, by examining the tan δ (tan δ = *G*″/*G*′, also known as the damping factor, is a parameter that describes the ability of a material to dissipate energy under vibration or dynamic loading), we can clearly identify the phase transition temperature, which is ≈45 °C, slightly above body temperature. According to the principle of time‐temperature equivalence, phase transitions that can be rapidly observed at 45 °C may take longer to manifest at 37 °C. Dynamic mechanical analysis revealed that both *G*′ and *G*″ exhibit similar trends, maintaining similar magnitudes and undergoing a phase transition at ≈42 °C (Figure 2b). Different methods were used to test the temperature‐dependent dynamic modulus of PAA/M‐HPMC hydrogel, and the results were consistent, demonstrating the reliability of the data and confirming that the hydrogel is responsive to temperature.

To further understand the relationship between force and strain during temperature variations in the gel, which is of utmost interest, we conducted additional experiments. Initially, under a strain of 1%, the static force gradually increased with rising temperature. At 37 °C, the static force reached 0.016 N, as shown in Figure 2c. When we started with an initial static force of 0.03 N, the strain gradually shifted from an initial 1.5% to negative values as the temperature increased. At 37 °C, the strain was −1.26%, indicating hydrogel contraction under these conditions, as shown in Figure 2d. These experiments quantified the force generated by hydrogel contraction at 37 °C, with force increasing alongside strain (a dynamic process).

### 2.3 In Vitro Antibacterial Activity and Biocompatibility

Antimicrobial wound dressings play a crucial role in preventing delayed wound healing caused by infections. To assess the antimicrobial efficacy of the hydrogels, we analyzed bacterial growth on agar and co‐cultivated different quality hydrogels with bacterial suspension. The optical density (OD) values of *E. coli* and *S. aureus* solutions decreased as the hydrogel quality increased (Figure S8a,b, Supporting Information). Additionally, the number of bacterial colonies showed a significant difference between the co‐culture of 50 mg mL^−1^ hydrogel with bacterial solution for 8 h and the control specimen (Figure S8c,d, Supporting Information). This led us to speculate that the antibacterial properties of skin stress‐shielding wound dressings were related to the release of Ag^+^. Hence, we performed the Ag^+^ release study and observed that the amount of Ag^+^ released increases over time (Figure S9, Supporting Information).

Given that the skin stress‐shielding wound dressing would directly interact with skin tissue, further research into the cytocompatibility of the hydrogels was necessary. We focused on fibroblasts, which play a vital role in wound healing, to assess the toxicity of the hydrogel. First, different quality stress‐shielding wound dressings were used to cultivate fibroblast cells for 1, 3, and 5 days. The OD value of each group increased with prolonged culture time (Figure S10a, Supporting Information). After 24, 72, and 120 h of culture, the in vitro biocompatibility of the stress‐shielding wound dressing was found to be comparable to the control media, with no statistical difference in cell viability for L292 fibroblasts (Figure S10b, Supporting Information). These results provide strong evidence supporting the tissue‐repairing potential and excellent biocompatibility of the stress‐shielding wound dressing.

### 2.4 In Vivo Wound Healing Efficacy in a Rat Model

**Figure 3:**
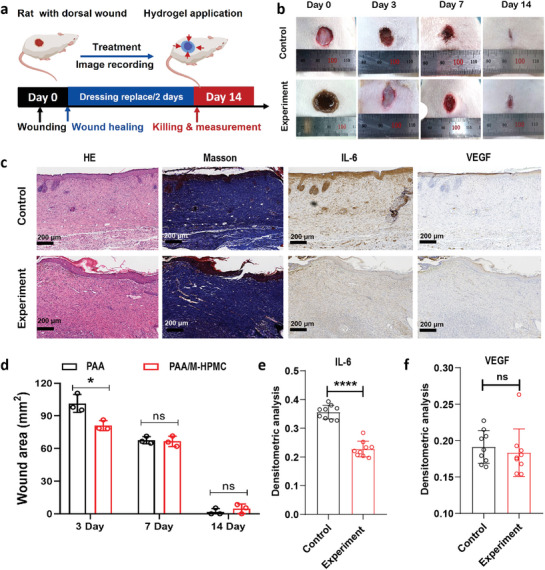
Evaluation of wound healing in a rat full‐thickness dermal wounds model. a) Schematic illustration of in vivo experiments design. b) Representative photographs of the rat wound closure on the 3rd, 7th, and 14th day (n = 3). c) Representative photos of the histomorphological and immunohistochemistry analysis of the skin tissue by H&E staining, Masson staining, IL‐6 and VEGF (n = 3). d) Statistical area of wound in healing process, n = 3. e) Semi‐quantitative statistics of IL‐6 levels n = 3. f) Semi‐quantitative statistics of VEGF levels, n = 3. Three section slices were taken from various rats. Additionally, three identically sized regions were selected from each slice for statistical evaluation of the expression of the proteins IL‐6 and VEGF. All data are shown as mean ± S.D.

We employed a well‐known full‐thickness skin damage model of wound healing in rats to evaluate the effectiveness of the stress‐shielding wound dressing in vivo. The full‐thickness lesions (10 mm in diameter) were created on the rat dorsal skin. Subsequently, the hydrogel wound dressing was applied to the wound to promote healing. **Figure** **3**a depicts a schematic diagram of the experimental procedure used in vivo to evaluate the ability of the hydrogel to promote wound healing. For 0, 3, 7, and 14 days, we captured the periodic photos of the rat wound sites to document the healing progress. Figure 3b displays the wound closure states for each group on the 3rd, 7th, and 14th days. In Figure S11 (Supporting Information), we have provided schematic diagrams depicting the dynamic healing of wound sites. Upon analyzing the statistics of wound area, we observed that the experimental group exhibited significantly smaller wound areas than the control group on the 3rd day. Surprisingly, on the 7th and 14th day, the rate of wound closure in the experimental group was no longer faster than that in the control group. In fact, the wound area of the control group was slightly smaller than that of the experimental group (Figure 3d). Indeed, the mechanical forces generated by temperature‐sensitive contraction dressings mainly affect the scar formation at the wound sites, which will be discussed in Section 2.5. Consequently, we also designed additional experiments to further explore the impact of skin stress‐shielding platform remodeling wound tension on the outcomes of skin wound healing.

The quality of the regenerated skin tissue was analyzed using Masson staining and hematoxylin and eosin (H&E) on the 14th day. The H&E and Masson staining images revealed the presence of granulation tissue, epidermis, a few hair follicles, and collagen deposition in both the experimental and control groups (Figure 3c). However, there appeared to be no significant difference in the information obtained from pathological tissue images between the experimental and control groups. Interleukin‐6 (IL‐6) has been associated with the inflammatory reaction and plays a role in the wound healing. Upon stimulation by IL‐6, macrophages, keratinocytes, and fibroblasts release vascular endothelial growth factor (VEGF). Therefore, we examined the expressions of IL‐6 and VEGF during the healing phase to assess the healing process. On the 14th day, the experimental groups that wore the hydrogel wound dressing exhibited weak positive staining for IL‐6, whereas the control group showed a significant positive result, indicating persistent inflammation in the untreated wounds (Figure 3c,e). Regarding VEGF expression, there was no discernible difference between the experimental and control groups (Figure 3c,f). This may be caused by vascular degeneration in the late stage of skin tissue repair.

### 2.5 Therapeutic Skin Scar Effects of a Skin Stress‐Shielding Wound Dressing

**Figure 4:**
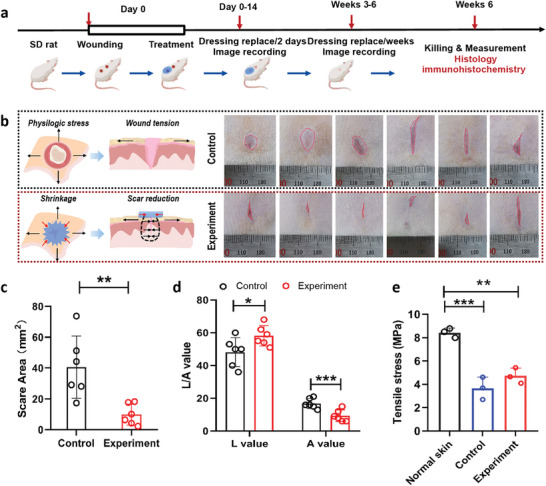
Evaluation of skin scar formation in the SD rat full‐thickness dermal wound model. a) Schematic illustration of the in vivo experiment design. b) Image of wounds on day 42 for the control and experimental groups. The hydrogel wound dressings were removed before imaging. (n = 6) c) Scar area of the differently treated groups. (n = 6) d) Color measurement of scars via the Photoshop Color Pickup Tool and LAB color model. The value of L represents a white color, and the value of A represents a red color. Values in (c) and (d) represent the mean ± S.D. (n = 6 for control groups and n = 6 for experimental groups). e) The tensile stress of skin specimens (n = 3 for normal skin, n = 3 for control groups and n = 3 for experimental groups).

To assess the efficiency of the skin stress‐shielding hydrogel wound dressing in vivo, we chose a full‐thickness damaged wound healing model. By applying the skin stress‐shielding hydrogel wound dressing onto dorsal excisional wounds with a diameter of 10 mm, it was found markedly reduced formation of skin scars at 6 weeks post‐injury, as evaluated by the area, color, and biomechanical properties of skin scars compared with the no stress‐shielding platform. **Figure** **4**a depicts a schematic diagram outlining the comprehensive experimental process. After 6 weeks of treatment, the appearance of scars is seen in Figure 4b. Statistical analysis demonstrated a considerable difference between the skin scars in the experimental and control groups, as illustrated in Figure 4c. The color of the newly healing tissue was evaluated using the Vancouver Scar Scale.^[^ ^21^ ^]^ Herein, the color model of LAB was used to characterize skin scars. Notably, the values of lightness (L: 58.2) and redness (A: 9.3) of the skin scars were remarkably close to those of normal skin, in contrast to the control scars, as shown in Figure 4d. These results indicate the effectiveness of the skin stress‐shielding hydrogel wound dressing in promoting improved wound healing and reduced scarring.

Prominent triggering factors that instigate the formation of skin scars during wound healing process encompass tension force and stress exerted on the skin. To obtain a more comprehensive understanding of the impact of stress‐shielding platforms on wound healing, an in‐depth analysis of the mechanical properties of both skin and skin scar tissue was conducted. Through an experiment involving uniaxial stress tensile failure, normal skin and scars underwent evaluation to determine their resistance to deformation and load‐bearing capabilities, as shown in Figure S12 (Supporting Information). Notably, we found that the force and elongation of scar tissue were inferior when compared to those of normal skin. The control skin scar specimen displayed the lowest tensile strength of 3.66 ± 0.65 MPa, in contrast to the experimental skin scar specimen, which displayed a tensile strength of 4.72 ± 0.47 MPa (Figure 4e). Despite exhibiting significant differences from normal skin, the extensibility and tensile strength of experimental scar tissue were augmented through the application of the stress‐shielding wound dressing. This indicated that the physical intervention of a skin stress‐shielding wound dressing resulted in an improvement of the tensile strength and coloration of the skin scar, while concurrently reducing the scar area.

### 2.6 Dressing Suppression of Skin Scar via Mechanical Remodeling

**Figure 5:**
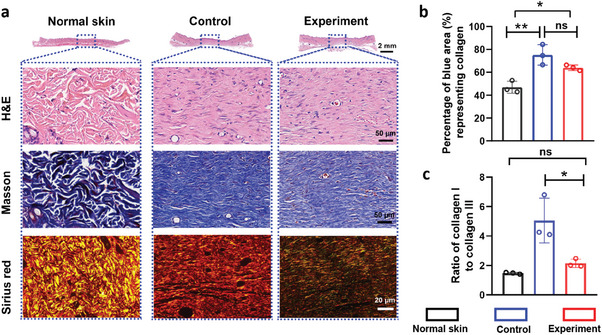
Histological analysis. a) Representative H&E, Masson and Sirius red staining images of scar tissue and normal skin, n = 3. b) Semi‐quantitative statistics of collagen, n = 3. c) The ratio of collagen type I (red) to type III (green). Three section slices were taken from various rats. Additionally, three identically sized regions were selected from three slices for statistical evaluation of the ratio of collagen I to collagen III. All data are shown as mean ± S.D.

To comprehensively assess the therapeutic effectiveness of skin stress‐shielding wound dressing, we further examined the pathological characteristics of scar tissue samples at 6 weeks post‐injury, focusing on excessive deposition of collagen and enhanced cellularity, which are key factors in scar formation. From section H&E staining, we observed a striking difference in cellularity and oriented cell arrangement compared to normal skin tissue (**Figure** **5**a). The control group tissue displayed increased cellularity and a more organized cell alignment. Masson staining showed that the deposition of collagen in the newly formed skin tissue was significantly higher than in normal skin tissue. Meanwhile, wound tissue treated with a stress‐shielding wound dressing had a lower density of collagen fibers compared with the control group (Figure 5b). The average percentage of collagen fibers in the Masson staining section was 46.92 ± 3.98%, compared to 75.13 ± 6.11% in the control group and 63.97 ± 1.64% in the experiment group, respectively. The deposition of total collagen in the wound increases with the progression of time during the entire healing process (Figure S13, Supporting Information). And there was significant variation in the morphology of collagen fibers at different time points (Figure S13, Supporting Information). Comparisons between the control and experimental groups revealed that the deposition of total collagen in the skin of rats treated with wound tension shielding dressings was slightly lower than that in the control group at different time points (Figure S14, Supporting Information). Additionally, Sirius red staining was used to examine type I (red) and type III (green) collagen in more detail. The analysis revealed two different ratios of type I (red) to type III (green) collagen in the tissues from the control group (≈5.05:1) and normal skin (≈1.47:1). In contrast, the ratio significantly decreased in the stress‐shielding wound dressing group, reaching 2.47 (Figure 5c). This demonstrated that type I collagen, which is often regarded as the most major component of the ECM of hypertrophic scars, was successfully blocked by the stress‐shielding wound dressing treatments.^[^ ^22^ ^]^ Overall, the dressing optimizes the ECM deposition and reduces the formation of skin scars during wound healing.

**Figure 6:**
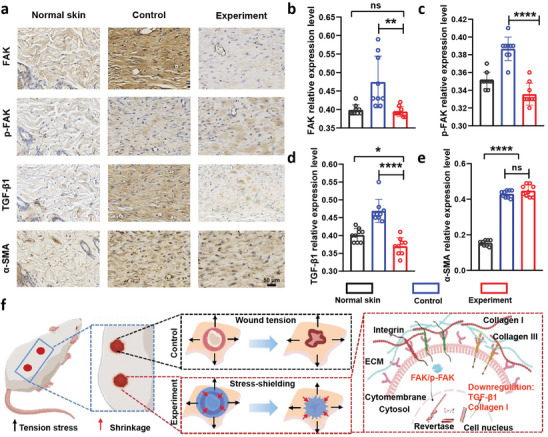
Immunohistochemistry staining analysis and illustration of the mechanical signaling pathway. a) Representative images of immunohistochemistry staining of FAK, p‐FAK, TGF‐β1 and α‐SMA. b–e) Semi‐quantitative statistics of FAK, p‐FAK, TGF‐β1 and α‐SMA protein levels. Three section slices were taken from various rats. Additionally, three identically sized regions were selected from each slice for statistical evaluation of the expression of the proteins FAK, p‐FAK, TGF‐β1 and α‐SMA. All data are shown as mean ± S.D. f) Illustration of stress‐shielding hydrogel wound dressing intervening the process of wound healing to reduce the skin scar.

FAK is a non‐receptor cytoplasmic tyrosine kinase that mediates the biomechanics signal pathway and takes part in mechanical signal transduction and fibroblast activation during the skin wound healing process.^[^ ^23^ ^]^ FAK is typically activated after skin damage, and the increased skin tension enhances the activation of FAK via phosphorylation. We used immunohistochemical labeling to assess the levels of these mechanotransduction‐related proteins in scar tissue in order to look into the potential involvement of FAK and p‐FAK in this pathway. **Figure** **6**a demonstrated that FAK staining was widely distributed throughout the scar tissue and displayed relatively high intensity in the control group compared to normal skin tissue. However, the expression of FAK was nearly absent in the experiment group treated with the stress‐shielding hydrogel dressing (Figure 6b). Additionally, the relative level of p‐FAK expression in the control group was also higher than those in normal tissue and the experiment group (Figure 6c). These findings suggest that the skin stress‐shielding hydrogel wound dressing effectively remolded the stress concentration around the wound by a mild physical intervention, leading to the suppression of FAK/p‐FAK expression and activation.

TGF‐β) is a crucial regulator that modulates fibroblast migration, proliferation, and ECM deposition, exerting pleiotropic effects on wound healing.^[^ ^24^ ^]^ Among the TGF‐β isoforms, TGF‐β1 is particularly important in skin tissue as it is a profibrotic cytokine that induces fibroblast‐to‐myofibroblast transformation. Activation of TGF‐β1 is potentiated by increasing wound tension and stress concentration. The expression of TGF‐β1 promotes excessive ECM deposition, leading to tissue fibrosis and macroscopic manifestations of skin scar hyperplasia. Figure 6a,d shows that the expression of TGF‐β1 was dramatically down‐regulated in the experimental group treated with the skin stress‐shielding hydrogel wound dressing compared to the control group. This result provides compelling evidence that the skin stress‐shielding hydrogel wound dressing can remold the biomechanical microenvironment of the wound to suppress skin scar formation.

Myofibroblasts are the activated phenotype of fibroblasts that promote collagen and ECM deposition during the process of wound healing.^[^ ^25^ ^]^ Myofibroblasts are significantly up‐regulated, contributing to contract wound and tissue remodeling, which are identified by staining with α‐SMA.^[^ ^26^ ^]^ Figure 6a,e demonstrates that the neonatal tissue had much higher levels of α‐SMA expression than normal skin tissue. This experimental result was not consistent with our ideal results. The persistent presence of highly expressed α‐SMA in healed wounds will lead to hypertrophic scars. According to the experimental results, despite the fact that the skin scars in the experimental groups were much less than those in the control group (Figure 4b), we discovered that the expression of a‐SMA was not significantly different (Figure 6e). The possible reason for this experimental phenomenon is that the biomechanical strength of the newborn skin tissue is not sufficient to offset the stress of the surrounding wound skin, resulting in the activation of a‐SMA. Also, this suggests that we need to extend the use time of the stress shielding wound dressing in future experiments.

Taken together, our results provide evidence that the stress‐shielding wound dressing significantly reduced the area of skin scars in rats after 6 weeks of treatment. Immunohistochemical staining of animal tissue samples treated with the dressing significantly down‐regulated the biomechanically mediated transducing signals, including FAK/p‐FAK expression and TGF‐β1. The schematic diagram in Figure 6f illustrates how the skin stress‐shielding wound dressing remolded the stress concentration around the wound, and having an impact on the formation of skin scars.

## 3 Conclusion 

In our study, we developed a hydrogel composed of M‐HPMC and PAA, which possessed temperature‐responsive contractility, strong adhesion to skin tissue, antibacterial properties, and excellent biocompatibility. This hydrogel was considered a promising candidate material for wound dressing applications. We demonstrated its ability to effectively remold stress concentration periwound, thereby reducing the formation of skin scar. The intervention mechanism was mainly to decrease wound tension, inhibiting the activation and transduction of fibroblasts, as well as excessive deposition of collagen. The low‐stress microenvironment created by the hydrogel dressing effectively blocked FAK/p‐FAK‐mediated mechanical signaling and the transforming growth factor TGF‐β1. Overall, the skin stress‐shielding wound dressing successfully re‐established a low‐stress microenvironment at the wound site, leading to a reduction in the skin scar area. This innovative approach represents a new strategy for high‐quality care and repair of skin wounds.

## 4 Outlook

Treating and preventing skin fibrosis is a crucial requirement within the biomedical field. Recent studies have revealed that mechanical force can cause fibrosis repair, and the significance of mechanical force in scar pathophysiology is becoming more widely recognized. Although surgical resection, steroid injection, laser therapy, and radiation therapy are commonly used in clinical practice, they often present challenges such as high cost, complexity, pain, or limited efficacy. Non‐invasive treatment methods, such as pressure clothing, silicone sheeting, and paper tape, have shown promising results, potentially attributable to their ability to manipulate mechanical forces. However, comprehensive research in this field remains limited. Our investigation, using a full‐thickness rat wound model, provides evidence supporting the effectiveness of the skin stress‐shielding wound dressing in reducing skin scars. In comparison to the control group, some wounds displayed a significant improvement in scar appearance, while others showed less noticeable improvement. Immunohistochemical staining analysis further demonstrated that the expression of growth factors associated with mechanical force induction was inhibited after using the skin stress‐shielding dressings. Nevertheless, there are still some areas that need further adjustment and improvement in this work. For instance, we did not look into how the duration of wearing the stress‐shielding dressings impacted the appearance of skin scars. In the complex and dynamic wound healing process, the timing of dressing intervention is also a clinical issue worth exploring in wound care. These aspects will be addressed in future research endeavors. Future research should concentrate on wound‐specific considerations, optimal duration for utilizing stress‐shielding platforms, and quantifying the contraction force of dressing and tension in wounds.

## 5 Experimental Section

### Materials

Unless otherwise specified, all chemicals were purchased from Aladdin Industrial Corporation. For preparation of the skin stress‐shielding platform, hydroxypropyl methyl cellulose (HPMC), acrylic acid (AA), sodium lignin sulfonate (SLS), ammonium persulfate (APS), silver nitrate (AgNO_3_), dopamine (DA), ferric chloride (FeCl_3_⋅6H_2_O), *N,N*‐methylene bisacrylamide (BIS), sodium hydroxide (NaOH), hydrochloric acid (HCl), α‐ketovaleric acid, glutaraldehyde, tris(hydroxymethyl)methyl aminomethane (Tris), allyl bromide, dimethyl sulfoxide (DMSO) and pyrrole (Py) were used. The Py was obtained from the Tokyo Chemical Industry. All porcine tissues used in vivo were purchased from a porcine tissue vendor.

### Preparation of the Skin Stress‐Shielding Hydrogel Wound Dressing

Hydroxypropyl methylcellulose (2% w/w) was dissolved in a solution of NaOH (1 m). Subsequently, allyl bromide (4% v/v) was added to the hydroxypropyl methylcellulose solution and allowed to react at room temperature for 24 h. The resulting reaction solution was then neutralized by adding HCl (1 m). After purification and freeze‐drying, high‐purity modified hydroxypropyl methyl cellulose (M‐HPMC) was obtained. And then, M‐HPMC (8% w/w) was dissolved in distilled water to get a precursor solution. Concurrently, acrylic acid (50% v/v) was dissolved in a DMSO/deionized water mixture (1:1, v/v). Next, APS (1% w/w) and PPy‐PDA (0.4% w/w) nanoparticles were added after the precursor solution and acrylic acid solution were mixed in a ratio of 1:2. Finally, a solution of Ag‐SLS (5% v/v) was added to the above mixture. To create the skin stress‐shielding hydrogel wound dressing, the mixed solution was applied to a mold and subjected to conditions at 60 °C for 20 min. The procedures for synthesizing PPy‐PDA and Ag‐SLS nanoparticles were based on earlier research.^[^ ^19^, ^20^ ^]^ For additional information on the preparation process of M‐HPMC and PAA hydrogel, please refer to the Supporting Information (SI).

### Characterization of Hydrogel Wound Dressing

The skin stress‐shielding platform was immersed in deionized water to eliminate the DMSO solvent. Subsequently, various measurements were conducted to explore its properties. Nuclear magnetic resonance (^1^H NMR) was used to explore the chemical composition of M‐HPMC. The chemical structure of PAA/M‐HPMC hydrogel was examined using Fourier transform infrared spectra (FTIR) and X‐ray photoelectron spectroscopy (XPS). Scanning electron microscope (SEM) were used to measure the microstructure of the hydrogels. More detailed information about the characterization methods can be found in Supporting Information.

### Cytotoxicity of the Hydrogel Wound Dressing

The cytotoxicity of the skin stress‐shielding platform was assessed by the viability of L929 fibroblasts. Briefly, fibroblasts were incubated in 96‐well plates (2 × 10^3^ cells/well) for 12 h to allow for cell attachment. Then, sterile hydrogels (5, 25, and 50 mg mL^−1^) were added to wells, and the cells were co‐incubated at 37 °C for 24, 72, and 120 h. Cell Counting Kit‐8 (CCK‐8) was used to test the cytotoxicity of hydrogel. Each group had six parallel samples. The statistical significance was assessed by Graph Pad software.

### In Vivo Wound Closure of a Full‐Thickness Wound Model in Rats

As previously reported, the influence of hydrogel wound dressing on wound healing was investigated. Each step of the rat skin wound healing investigation received ethical approval from the animal ethics committee at West China Hospital, Sichuan University. The procedure involved administering pentobarbital anesthesia (20 mg kg^−1^ body weight) to the rats, followed by the creation of a circular wound with a diameter of 10 mm. Subsequently, the wound was treated with a hydrogel and replaced every two days. The progression of the wounds was observed and documented at specific time points, namely, 0, 3, 7, and 14 days. After 14 days, skin samples from the injured section were collected and fixed using 4% formaldehyde. The skin samples were then covered in paraffin wax for cutting into slices for analysis.

### To Assess the Effectiveness of Skin Stress‐Shielding Platform for Reducing Skin Scarring

All procedures involving the rat skin wound healing investigation were approved by the animal ethics committee of West China Hospital, Sichuan University. Two circular wounds, each with a 10 mm diameter, were made on dorsal area of the rats by removing full‐thickness skin after anesthesia with pentobarbital (20 mg kg^−1^ body weight). In the experimental group, the wounds were covered with a skin stress‐shielding hydrogel wound dressing. As for the control group, conventional gauze dressings were used to manage the wounds. For the initial 14 days, the dressings were changed every two days. After this period, the dressings were changed once a week. The wounds were monitored photographically regularly for 0–42 days. On days 42, six rats from each group were euthanized, and the wound skin was removed to facilitate further analysis.

### Biomechanical Testing

The mechanical test skin specimens were carefully collected along the axial axis of scar using a scalpel. The length, thickness, and width of the skin scar specimens were measured by a vernier caliper. The ends of the specimens were clamped by a mechanical testing machine. A force–displacement curve was recorded at a constant rate of 10 mm min^−1^. The specimens were stretched until failure, and ultimate strength was measured. To assess the colors of the scars, measurements were taken using the Photoshop Color Pickup Tool, with the LAB color model used for analysis.

### Histology, Immunohistochemistry Assays

Rat wound tissue samples were collected on days 14 and 42 by bisecting the wound center. The animal tissue specimens were then fixed in 10% formalin, dehydrated, and prepared for paraffin embedding. 5 mm thick sections were used for further analysis. Subsequently, staining procedures including Hematoxylin and eosin (H&E), Masson's trichrome and Sirius red were performed according to established protocols at the Institute of Clinical Pathology, West China Hospital, Sichuan University. Whole‐slide images were acquired using a tissue section scanner and polarizer accessories. To perform quantification analysis, Image J software was employed to count the positive area and normalize it to the overall tissue area.

For the immunohistochemistry assay, the paraffin‐embedded animal wound sections were processed as previously described. And then, tissue sections were incubated with primary antibodies against IL‐6 (Abcam, ab9324), VEGF (Abcam, ab1316), FAK (Thermofisher, 34Q36), p‐FAK (Abcam, Tyr576), α‐SMA (Abcam, 246 987) TGF‐β1 (Abcam, ab215715) diluted in blocking solution overnight at 4 °C. Following this step, sections were subjected to routine procedures for conjugating secondary antibodies and developing them. Using Image J, the proportion of targeting markers was calculated to further analysis.

### Statistical Analysis

GraphPad Prism was used for all statistical significance analysis in this work. One‐way analysis of variance (ANOVA) was appropriate for comparison between multiple data groups. Two‐sided t‐test was used for comparison between two data groups. The threshold values of ^*^ *P* < 0.05, ^**^P ≤ 0.01, ^***^P ≤ 0.001, and ^****^P ≤ 0.0001 were considered a significant difference between groups.

## Conflict of Interest

The authors declare no conflict of interest.

## Author Contributions

Q.C. conceived, devised, executed the experiments, and authored the initial draft with suggestions from W.Z., C.Z., and K.L. S.L. conducted the antibacterial, cellular, and animal experiments with guidance from Q.C. W.Z. revised, corrected, and performed the manuscript and project supervision.

## Supporting information



Supporting Information

Supplemental Movie 1

Supplemental Movie 2

## Data Availability

The data that support the findings of this study are available from the corresponding author upon reasonable request.
