# Supplementary material for: A Skin Stress Shielding Platform Based on Body Temperature‐Induced Shrinking of Hydrogel for Promoting Scar‐Less Wound Healing
Source: Adv Sci (Weinh). 2024 Sep 16;11(41):2306018. doi: 10.1002/advs.202306018 (PMC11538717; doi:10.1002/advs.202306018)
Supplement: Supplementary file 1 — Supporting Information [file ADVS-11-2306018-s001.docx]

***Supporting Information***

**A** **skin stress shielding platform based on body temperature-induced shrinking of hydrogel for promoting** **scar-less wound healing**

*Qin Chen^1,2^, Siyu Li^1^, Ka Li^2^, Weifeng Zhao^1,3*^, Changsheng Zhao ^1,3^*

^1^ College of Polymer Science and Engineering, State Key Laboratory of Polymer Materials Engineering, Sichuan University, Chengdu, 610065, China.

^2^ West China Hospital, Sichuan University/West China School of Nursing, Sichuan University, Chengdu 610041, China.

^3^ Med-X Center for Materials, Sichuan University, Chengdu, 610065, China.

* Corresponding author

E-mail: zhaoscukth@163.com; weifeng@scu.edu.cn (Weifeng Zhao)


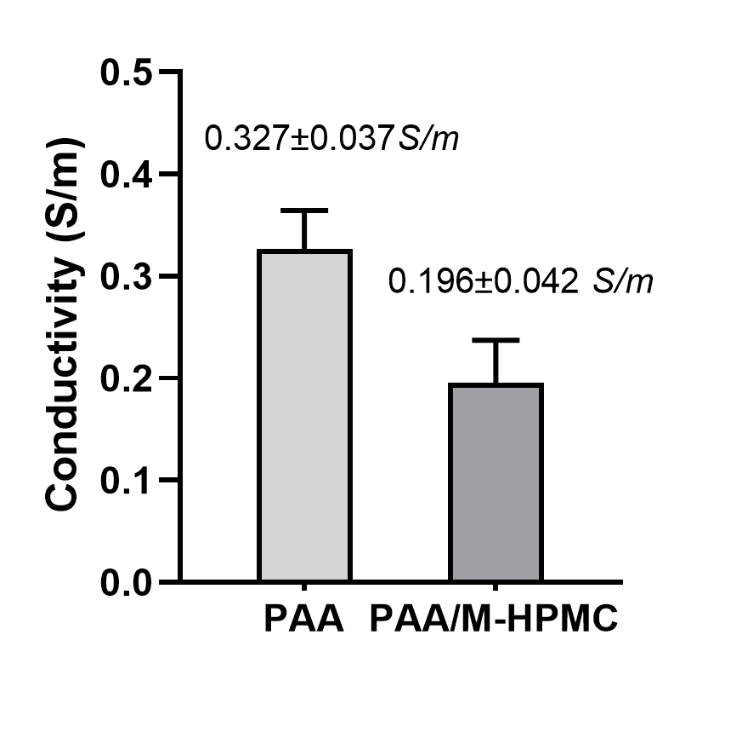


**Figure S1.** The conductivity of PAA and PAA/M-HPMC hydrogels.

The conductivity (δ) of the hydrogels is measured by a two-point method and calculated using the equation,

δ=L/RS eq. (1).

where L and S are the length and cross-sectional area of the sample, and R is the electrical resistance of the sample, respectively. Through testing, we found that the conductivity of PAA and PAA/M-HPMC hydrogels is 0.327±0.037 and 0.196±0.042 S/m respectively (see **Figure S1**).





**Figure S2**. ^1^H NMR spectrum of HPMC and M-HPMC.


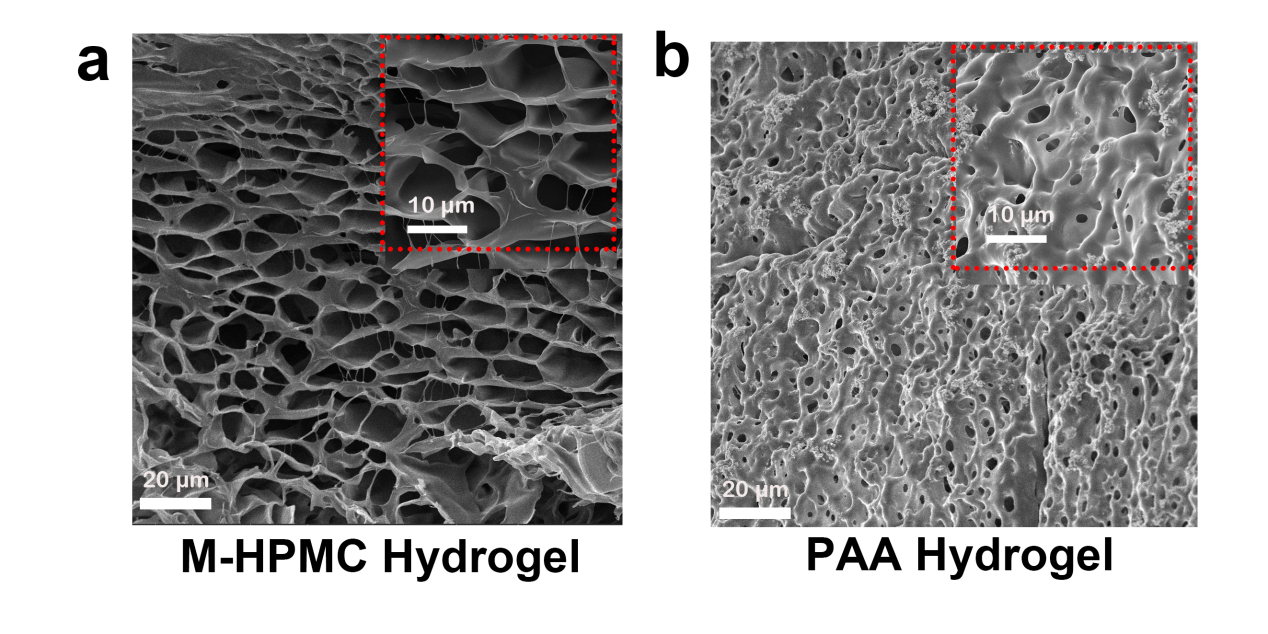


**Figure S3**. SEM images of (a) M-HPMC hydrogel and (b) PAA hydrogel.


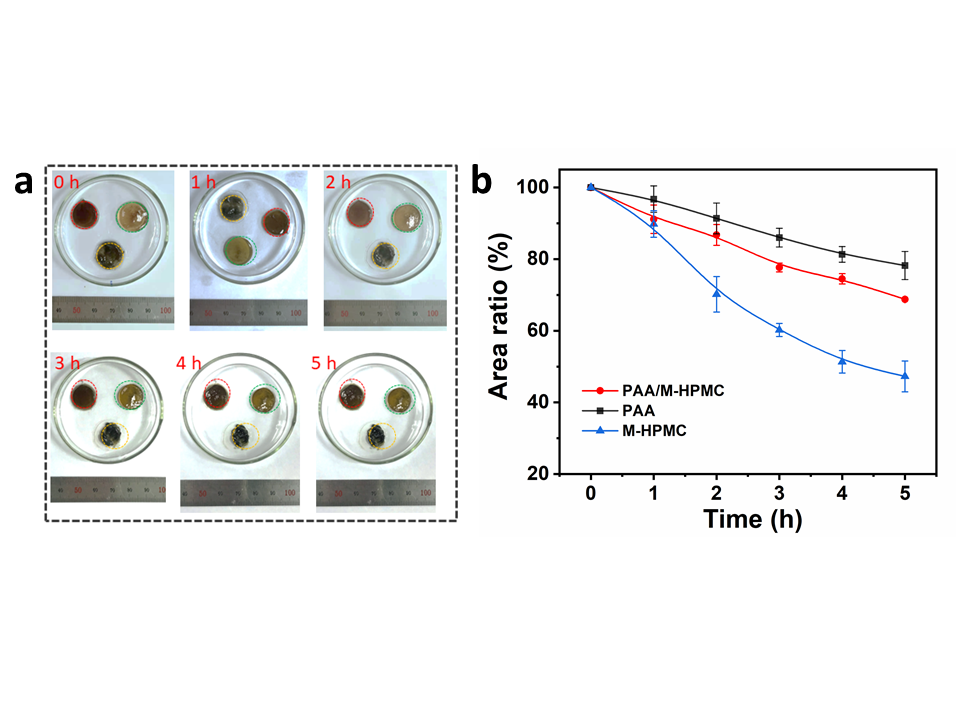


**Figure S4**. Photos of area shrinkage of M-HPMC hydrogel, PAA hydrogel, and PAA/M-HPMC hydrogel at 37°C. In these pictures, the red circle represents PAA/M-HPMC hydrogel, the green circle represents PAA hydrogel, and the yellow circle represents M-HPMC hydrogel.


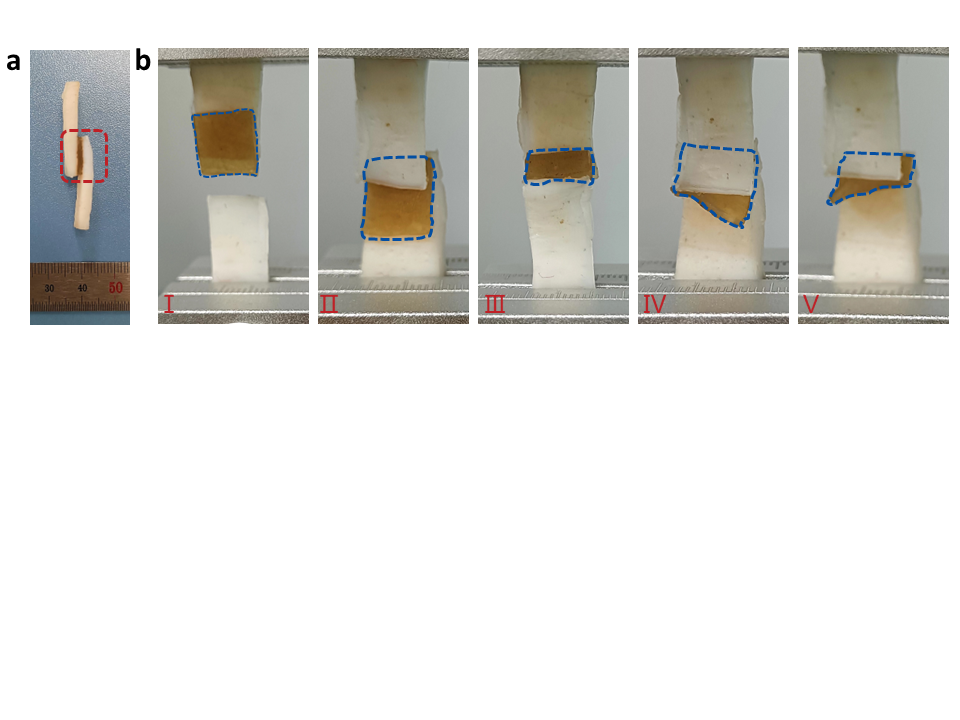


**Figure S5**. (a) Image of the hydrogel lap-shear experiment. (b) Images of the PAA/M-HPMC hydrogel sample after each adhesion stretching.


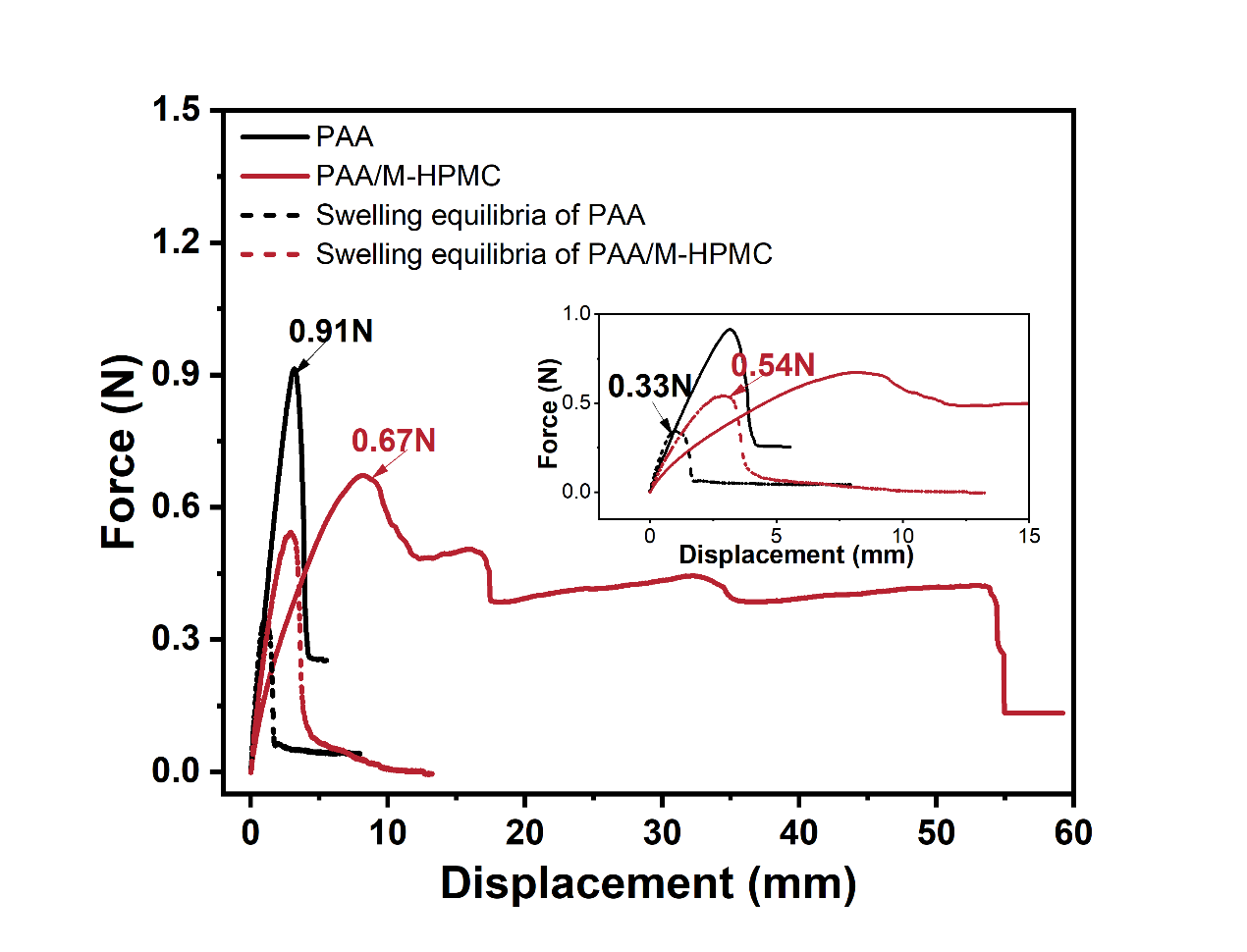


**Figure S6**. Displacement-Force plot before and after the swelling of PAA and PAA/M-HPMC hydrogel.


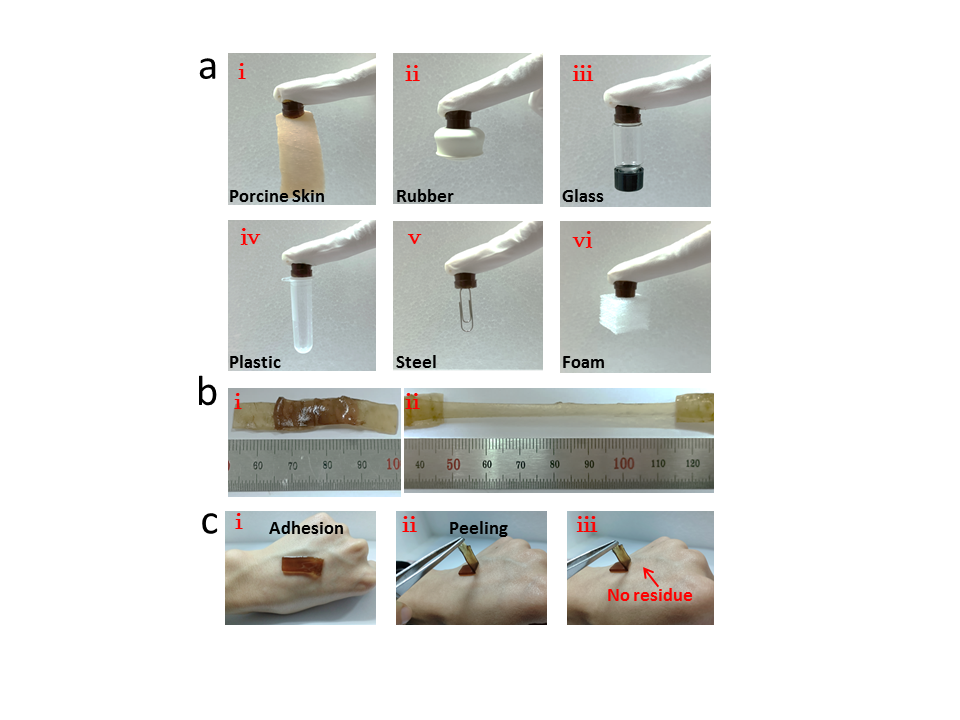


**Figure S7**. Photographs of (a) the adhesion property of the PAA/M-HPMC hydrogel on different substrates; The PAA/M-HPMC hydrogel could adhere to (b) porcine skin and produce certain deformation without falling off (c) PAA/M-HPMC hydrogel sticks to human skin.


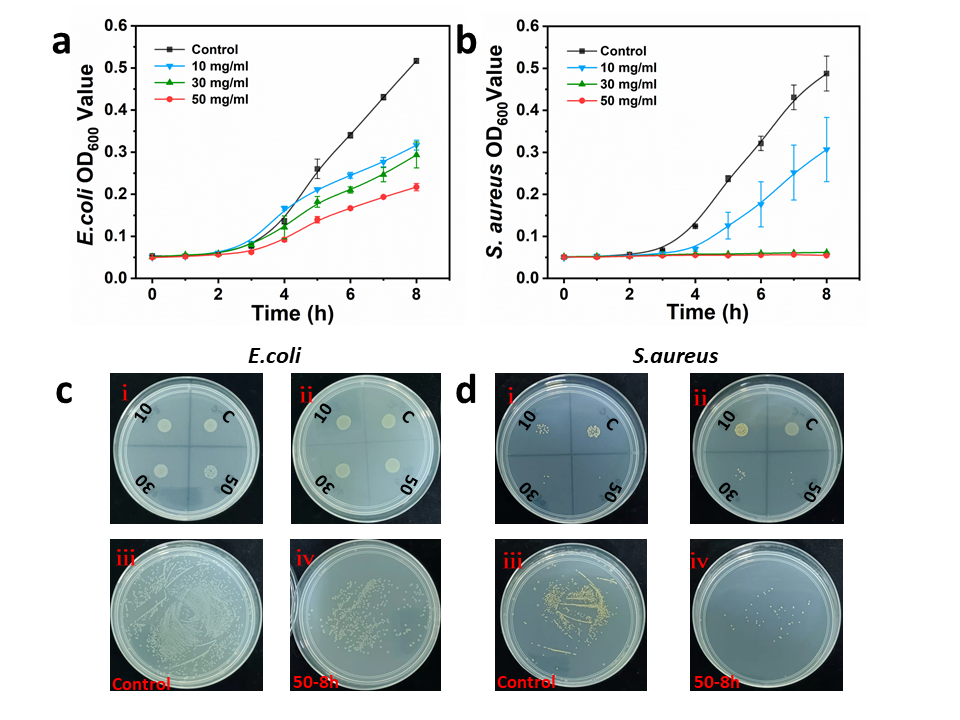


**Figure S8**. Time-OD values of (a) *E.coli* and (b) *S.aureus* bacteria suspensions co-cultured with PAA/M-HPMC hydrogel; (c) *E.coli* and (d) *S.aureus* bacteria suspensions were co-cultured with the hydrogel for a period and plated.





**Figure S9**. Cumulative Ag^+^ release from PAA/M-HPMC hydrogel at different time intervals.


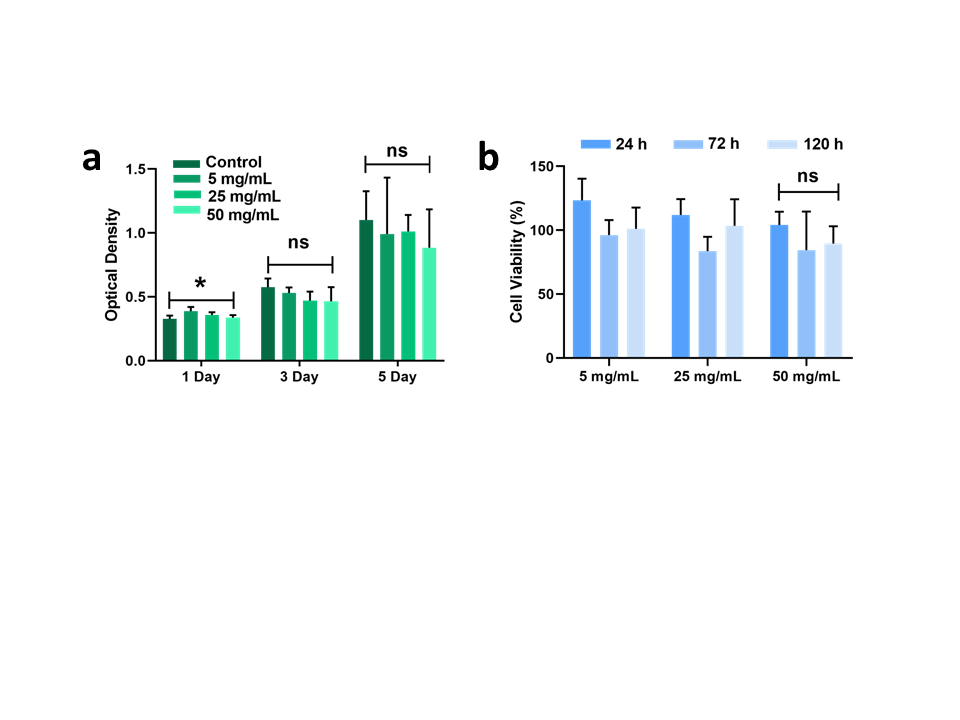


**Figure S10**. Cytocompatibility of the PAA/M-HPMC hydrogels: (a) The OD values of L929 fibroblasts cultured with hydrogel for 1, 3, and 5 days; (b) Cell viability of L929 fibroblasts after 24, 72, and 120 h of culturing with hydrogels.


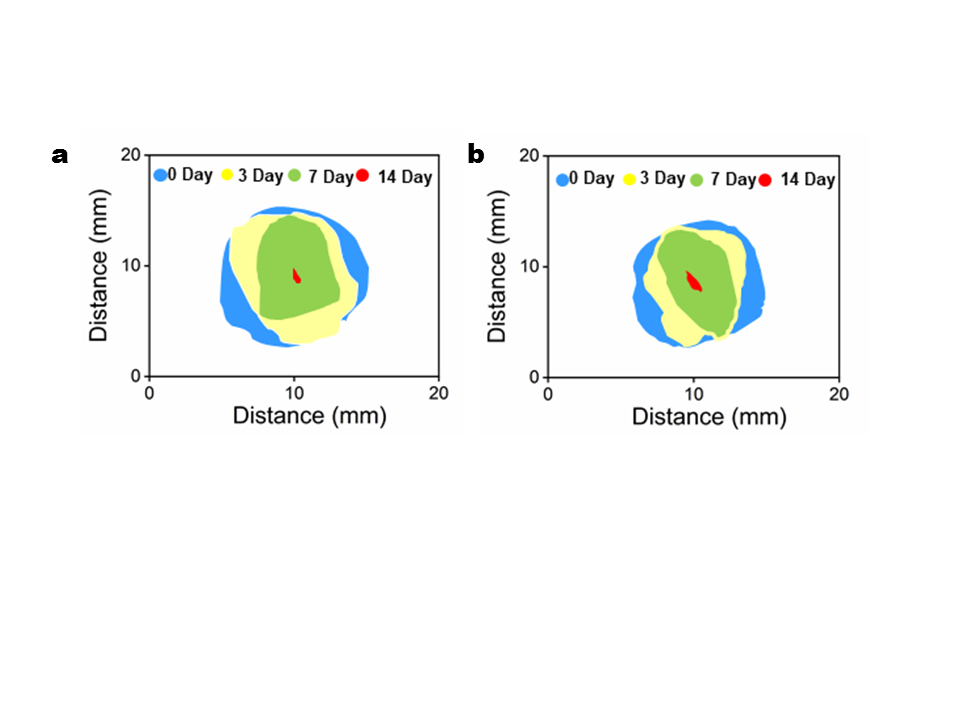


**Figure S11**. The schematic diagram of the wound area at different stages: (a) control group (b) experiment group.


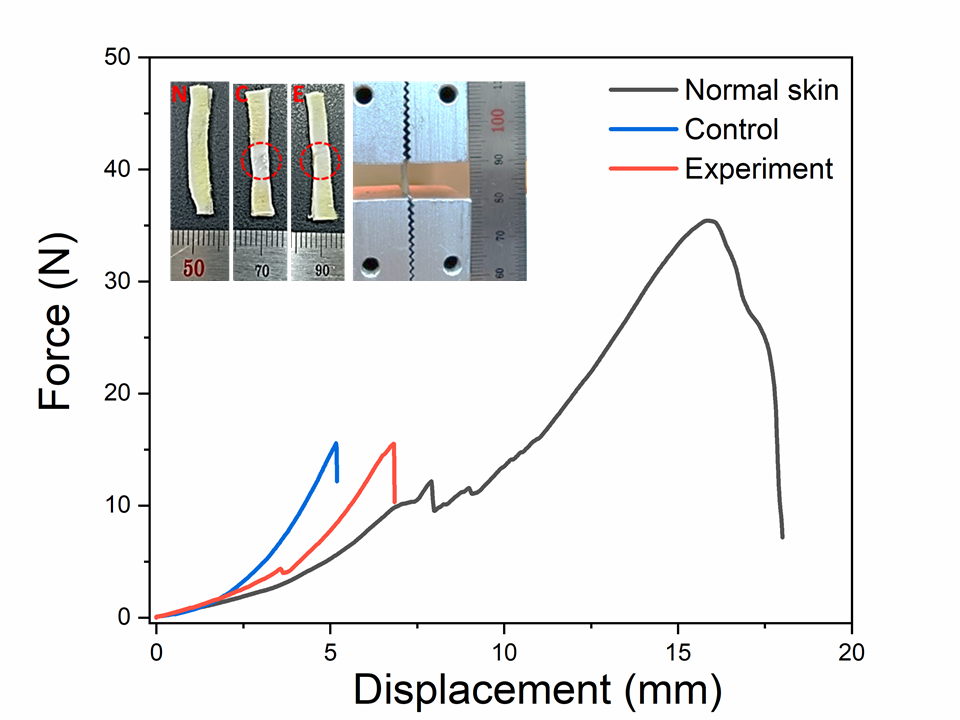


**Figure S12**. Representative curve of tensile skin experiments.


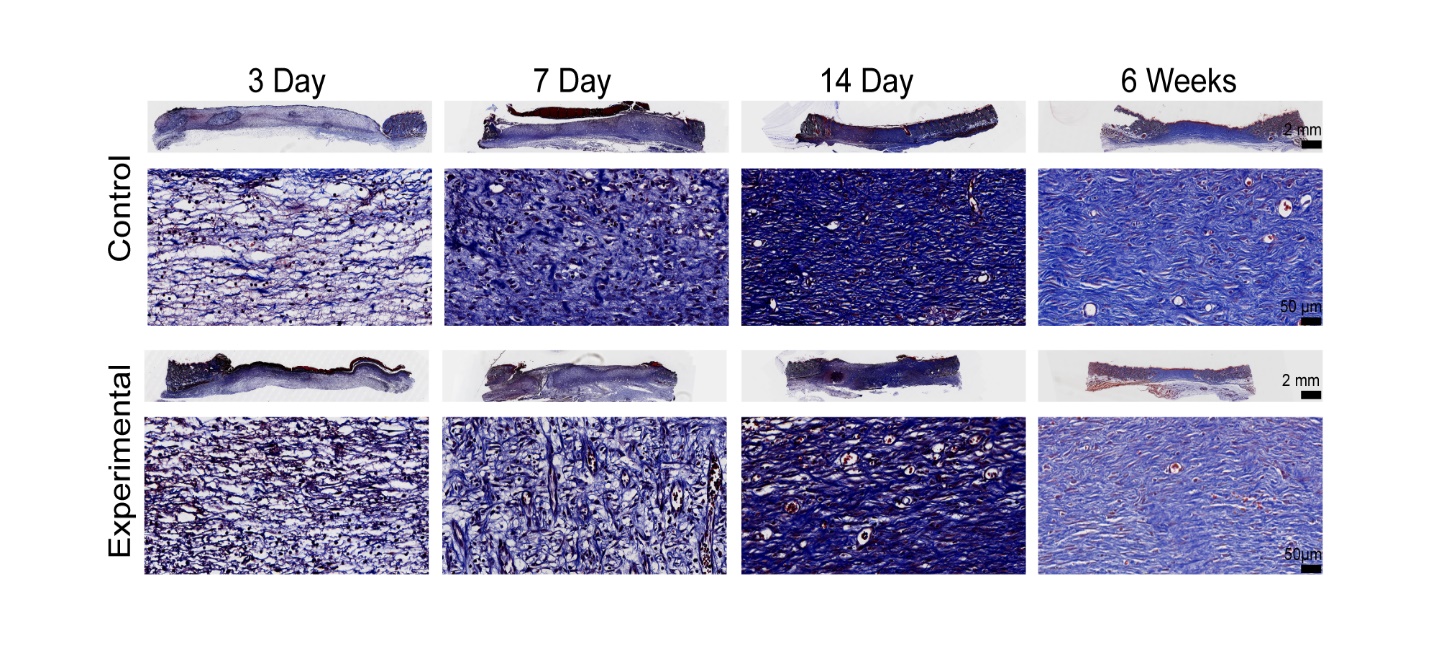


**Figure S13**. Representative photos of the histomorphological analysis of the skin tissue by Masson staining.


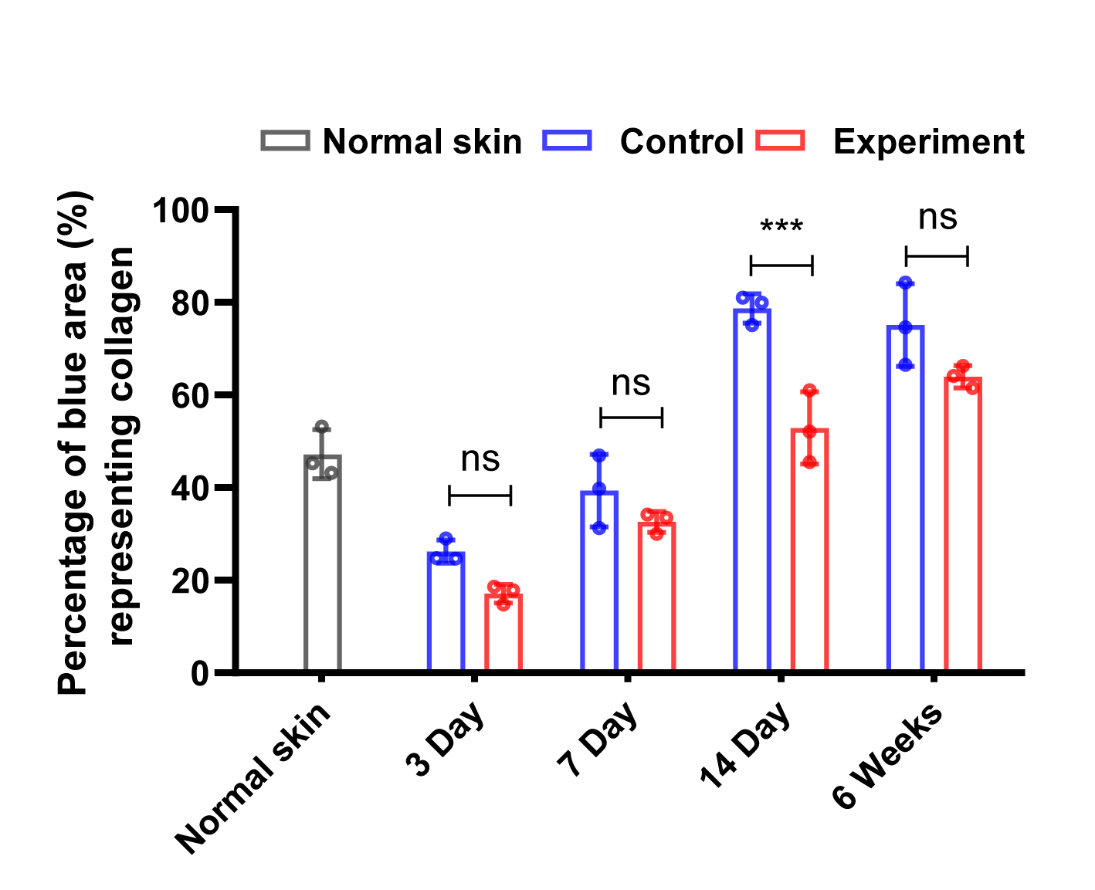


**Figure S14**. Semi-quantitative statistics of the total collagen deposition at different stages of the experiment.

***Preparation of the M-HPMC hydrogel.*** The high-purity modified hydroxypropyl methyl cellulose (M-HPMC) was obtained by purification and freeze-drying. And then, M-HPMC (8% w/w) was dissolved in deionized water to get a precursor solution. Next, α-ketovaleric acid (1% w/w), glutaraldehyde (2% w/v), and PPy-PDA (0.4 % w/w) nanoparticles were added after the precursor solution. Finally, Ag-SLS (5% v/v) solution was added to the above precursor solution, which was poured onto the mold to form a hydrogel under UV light for 30 min.

***Preparation of the PAA hydrogel.*** Acrylic acid (30% w/w) was dissolved in a NaOH (1 M) solution. And then, APS (1% w/w) and PPy-PDA (0.4 % w/w) nanoparticles were added after mixing the solution. Finally, Ag-SLS (5% v/v) solution was added to the above-mixed solution, which was poured onto the mold to form a hydrogel under vacuum (60°) for 20 min.

***Nuclear magnetic resonance (NMR).*** ^1^H NMR (400 MHz) spectra of HPMC and M-HPMC were tested by employing a Bruker AV III HD 400 MHz NMR instrument. DMSO was used as the solvent for HPMC and M-HPMC.

***Fourier transform infrared spectrometry (FTIR).*** The FTIR analysis was used to analyze the chemical groups of PAA/M-HPMC, M-HPMC, and PAA hydrogel materials. The spectra were collected at regions between 4000 and 800 cm^−1^.

***X-ray photoelectron spectroscopy (XPS).*** The elements of the hydrogel were determined with XPS analysis. The XPS spectra were recorded on an AXIS Supra instrument. Before the analysis, the hydrogel samples were freeze-dried.

***Scanning electron microscopy (SEM).*** The hydrogel morphology characterization was carried out using a SEM instrument (Thermo Fisher Scientific, Apero S Hivoc) at an accelerating voltage of 10 kV. Before the analysis, the hydrogels were freeze-dried.

***Adhesion tests.*** The adhesive properties of the hydrogels were determined using the lap-shear experimental method. The hydrogels were applied to the surface of the porcine skin (simulated human skin) with a bonded area of 10 mm×10 mm. The samples were pulled to failure at a speed of 10 mm/min until the samples separated. The shear strength was calculated by the maximum load divided by the initial bonded area. Five cyclic tests for each sample are examined. Three samples of each type.

***Characterization of hydrogel swelling behavior***. Immerse hydrogels of a certain mass of PAA and PAA/M-HPMC into a simulated body fluid (SBF) solution. At regular intervals, take out the gels, blot the surface solution using filter paper, and weigh the hydrogels. When the hydrogel mass no longer increases, it is considered to have reached swelling equilibrium.

***Rheological Experiments***. The viscoelastic behavior of PAA/M-HPMC hydrogels was characterized using a rotational rheometer (TA AR1500ex) at temperatures ranging from 25°C to 60°C. The storage modulus ($G^{'}$), loss modulus ($G^{''}$), and loss tangent of the hydrogel were determined in the frequency of 1 Hz at 1.0% strain amplitude.

***Dynamic thermomechanical analysis.*** The stress relaxation performance was conducted using a tension mode from 25 to 60℃ at a frequency of 1 Hz by the dynamic thermomechanical analysis (DMA, TA Q800). Rectangular specimens with a dimension of 17.7 mm×5 mm×1.8 mm were stretched to a strain of 1% at a strain rate of 10%/min, and then samples were held at the strain of 1% while the stress relaxation occurred. Alternatively, apply a fixed force of 0.03 N and observe the strain behavior of the material as the temperature increasing.
